# SIRNA-Directed *In Vivo* Silencing of Androgen Receptor Inhibits the Growth of Castration-Resistant Prostate Carcinomas

**DOI:** 10.1371/journal.pone.0001006

**Published:** 2007-10-10

**Authors:** Daniel Compagno, Carole Merle, Aurélie Morin, Cristèle Gilbert, Jacques R. R. Mathieu, Aline Bozec, Claire Mauduit, Mohammed Benahmed, Florence Cabon

**Affiliations:** 1 CNRS, University Paris-Sud, FRE2944, Epigenetics and Cancer, Institut André Lwoff, Villejuif, France; 2 INSERM U406, Lyon, France; University of Florida, United States of America

## Abstract

**Background:**

Prostate carcinomas are initially dependent on androgens, and castration or androgen antagonists inhibit their growth. After some time though, tumors become resistant and recur with a poor prognosis. The majority of resistant tumors still expresses a functional androgen receptor (AR), frequently amplified or mutated.

**Methodology/Principal Findings:**

To test the hypothesis that AR is not only expressed, but is still a key therapeutic target in advanced carcinomas, we injected siRNA targeting AR into mice bearing exponentially growing castration-resistant tumors. Quantification of siRNA into tumors and mouse tissues demonstrated their efficient uptake. This uptake silenced AR in the prostate, testes and tumors. AR silencing in tumors strongly inhibited their growth, and importantly, also markedly repressed the VEGF production and angiogenesis.

**Conclusions/Significance:**

Our results demonstrate that carcinomas resistant to hormonal manipulations still depend on the expression of the androgen receptor for their development *in vivo*. The siRNA-directed silencing of AR, which allows targeting overexpressed as well as mutated isoforms, triggers a strong antitumoral and antiangiogenic effect. siRNA-directed silencing of this key gene in advanced and resistant prostate tumors opens promising new therapeutic perspectives and tools.

## Introduction

AR is a ligand-activated transcription factor that plays a pivotal role in the prostate, regulating a large set of genes involved in cell division, apoptosis, and angiogenesis. Prostate carcinomas are initially androgen-dependent (ADCaP) and hormone ablation efficiently inhibits their growth, although some deleterious side effects result from the inhibition of AR signaling in normal tissues. The major limitation of these treatments is the capacity of tumors to escape and recur [1]. Once tumors became resistant to castration, the prognosis is poor. Non-specific and toxic chemotherapies like taxotere are only palliative, and the issue is usually fatal in less than 2 years. Increasingly, clinical observations demonstrate that in the majority of castration-resistant prostate carcinomas (CRCaP, also called hormone-refractory), AR is expressed and controls transcription. Although hormone ablation treatments strongly reduce the circulating testosterone levels, the residual amounts of androgens measured within the tumors of castration-resistant patients, are sufficient to maintain some AR signaling [2], [3]. Furthermore, several other processes allow CRCaP bypassing hormonal ablation [4], [5]. In experimental tumors, a moderate overexpression of AR in androgen-dependent tumors allows experimental tumors bypassing hormone ablation [6]. In patients, AR overexpression can result from gene amplification, increased transcription, or stabilization of the AR protein [7], recently linked to the phosphorylation of some AR residues [8], [9]. Other molecular events, such as mutations broadening the ligand spectrum, or conferring agonist properties to androgen antagonists, alterations in nuclear receptor coactivators, ligand-independent binding of AR to DNA, were also implicated in AR signaling in CRCaP [4], [5]. The variety of mechanisms that can be selected by prostate tumor cells to escape androgen-ablation therapies underlines their plasticity, and suggests that AR expression is mandatory in CRCaP. However, molecular alterations continue to accumulate once resistance to hormone ablation is established [10], and a fraction of aggressive tumors, like the DU145 and PC3 cells, stably silence AR, activate other signaling pathways, and become truly androgen-independent [11]. Thus, although AR is functional in most recurrent tumors [5], [12], disrupting AR signaling once the resistant phenotype is established, may not be sufficient to halt the tumor growth. Studies set up, in cultured cells, to test this hypothesis lead to contradictory conclusions [13], [14], [15], [16], [17]. Using RNA interference to study *in vivo* the role of AR in CRCaP, we demonstrate here that tumors that escaped hormonal manipulations are still dependent on the androgen receptor for their *in vivo* growth: AR silencing in tumors inhibits cells' proliferation, induces apoptosis and inhibits angiogenesis. Moreover, we establish the efficiency, safety and specificity of synthetic siRNA to treat those advanced tumors.

## Results

### Silencing of AR in ADCaP

**Figure 1:**
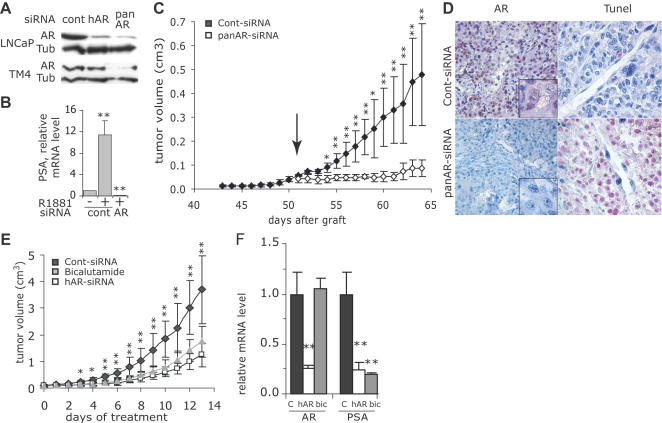
Silencing of AR in LNCaP cells and tumors. A: Control (cont)- panAR- or hAR-siRNA were transfected into human LNCaP or into mouse Sertoli TM4 cells. AR was immunodetected by western blot in cell lysates 2 days after removal of transfection medium. α-tubulin (tub) expression was used as a loading control. B: Relative PSA mRNA level in LNCaP cells transfected with control or hAR-siRNA and grown for 48 h in the absence of androgens or in the presence of R1881, 0.5 nM (mean±SE, n = 3 independent experiments). Similar results were obtained using the panAR-siRNA. **p<0.01 as compared to values in the absence of androgens. C: LNCaP cells were subcutaneously injected on day 0 to nude mice. Starting from day 51 (arrow), animals (5 per group) received a daily i.p. injection of 3 µg of cont- (black symbols) or panAR-siRNA (white symbols) diluted in 50 µl saline; tumor volume (cm^3^, mean±SE, *n* = 5). *p<0.05 and **p<0.01 comparing panAR-siRNA to cont-siRNA treated tumors. D: Analysis of AR expression by immunohistochemistry (left panels) and apoptotic cells by TUNEL (right panels) in representative tumors collected at the end of the experiment shown in C. E: Mice bearing exponentially growing LNCaP tumors were randomized (12 mice per group) and received daily i.p. injections of cont-siRNA (black symbols), hAR-siRNA (white symbols) or an oral dose of 50 mg.kg^−1^ of bicalutamide (grey symbols); tumor volume (cm^3^, mean±SE). *p<0.05 and **p<0.01 comparing hAR-siRNA to cont-siRNA treated tumors. F: On the fourth day of treatment of the experiment shown in E, 6 mice in each group were sacrificed and AR and PSA mRNA levels were quantified in the tumors by qRT-PCR and normalized with cyclophilin A mRNA level. Results are expressed relative to the mean level in control tumors. **p<0.01 comparing hAR-siRNA to cont-siRNA treated tumors.

We used in this study RNA interference to investigate *in vitro* and *in vivo* the function of AR in prostate carcinomas. To establish the technical conditions and specificity of AR silencing, we first used the human androgen-dependent prostate tumor model LNCaP. Androgens stimulate LNCaP cells' proliferation whereas castration and the androgen antagonist bicalutamide inhibit the development of xenografted LNCaP tumors in mice [18]. We designed and synthesized two different siRNAs targeting the first exon of AR. The panAR-siRNA targets a sequence conserved between the human and mouse AR mRNAs. It silences AR expression in the mouse Sertoli TM4 as in the human LNCaP cell line (Figure 1A). In contrast, the hAR-siRNA, which targets the human sequence but presents 5 mismatches out of 19 with the mouse mRNA, inhibits AR expression in LNCaP but not in mouse TM4 cells (Figure 1A). Transfection of AR-siRNA in LNCaP cells strongly inhibits the androgen-induced transcription of Prostate Specific Antigen (PSA), a prototypic AR-target gene (Figure 1B).

To set up the technical conditions to silence AR expression *in vivo*, we implanted LNCaP cells subcutaneously into male nude mice. Tumors started to develop after 45 days. Once the measurement of tumors for 5 days demonstrated their exponential growth (Figure S1), the mean volume of tumors was 49.7±8.6 mm^3^ (mean±SEM). Mice were then randomized for treatment and received daily for 2 weeks an intraperitoneal injection (i.p.) of 3 µg (125 µg.kg^−1^) of unmodified synthetic siRNAs diluted in saline. Half of the mice were treated with the panAR-siRNA, the other half with a control-siRNA (cont-siRNA) matching no known mRNA sequences in mouse and human databanks. The growth of tumors treated with AR-siRNA was very rapidly arrested, and after 10 days, the mean tumor volume represented only 15% of the control (Figure 1C). The nuclear AR labeling observed in cont-siRNA treated tumor cells was no longer detected in tumors treated with AR-siRNA (Figure 1D, left panel), where large areas of dead, TUNEL-positive cells were present (Figure 1D, right panel).

Similarly, the hAR-siRNA also inhibited the LNCaP growth in vivo, as efficiently as the AR antagonist bicalutamide (Figure 1E). In this experiment, the mean tumor volume on the first day of treatment was 109.2±30.2 mm^3^ (mean±SEM). Half of the animals in each group were sacrificed on the fourth day of treatment. At that time, the Prostate Specific Antigen (PSA) mRNA was strongly repressed in the hAR-siRNA and in the bicalutamide groups, while AR mRNA level was repressed only in the hAR-siRNA treated tumors (Figure 1F).

### Specificity of the AR-siRNA induced inhibition of tumor growth

The weight and behavior of mice were not affected by a 3 weeks-long daily treatment with siRNAs, whatever the sequence used. After dissection, all organs were carefully examined and appeared normal with no sign of necrosis. TUNEL labeling did not reveal apoptosis of any cell type in the liver, including endothelial cells. Therefore, no toxic effects were observable on a macroscopic level.

Off-targets effects of siRNA, due to their partial complementarity with mRNA unrelated to the cognate sequence, are almost impossible to rule out by sequence analysis and siRNA design. Rescue experiments are inadequate here, because even a modest increase in AR expression modifies the phenotype of the prostate tumor cells [6]. Because off-target effects occur independently of the target [19], we injected AR-siRNA into mice bearing tumors that do not express AR. We chose the fibrosarcoma JT8 cell line for this purpose, because we previously demonstrated that injections of VEGF-siRNA into mice bearing JT8 tumors inhibited by 70% the VEGF production, resulting in a sustained inhibition of the tumor growth [20]. In contrast with the VEGF-siRNA, AR-siRNA did not inhibit the growth of JT8 tumors (Figure S2).

Long dsRNA and viruses activate the interferon/RNAseL pathway, triggering a non sequence-specific degradation of cellular RNAs and apoptosis [21]. Naked siRNA, even at a 2.5 mg/kg regimen, do not activate the interferon pathway [22], but some authors, using lipid-formulated siRNAs [23], reported such effects, possibly through the Toll-like receptors, TLR3, TLR7 or TLR8 [23], [24]. We therefore quantified the IFN-α protein level in the serum, and the interferon β mRNA level in the tumors, spleens, or livers, of mice treated for 3 weeks with panAR-siRNA, hAR-siRNA or cont-siRNA. None of the siRNA significantly modified these levels (Figure S3 and data not shown). In addition, it is noteworthy that RNAseL/HPC1 is one of the major susceptibility genes identified in familial prostate cancers [25]. Germline and sporadic mutations of RNAseL are found in the majority of prostatic tumors, including LNCaP cells, and result in a reduced enzymatic activity and capacity of cells to respond to the activation of interferons, likely favoring the development of prostate tumors [25]. It is therefore highly unlikely that the inhibition of LNCaP tumor growth observed in AR-siRNA treated mice results from a non-specific mRNA degradation and apoptosis induced by interferons.

### Silencing of AR in prostate and testes of treated mice

**Figure 2:**
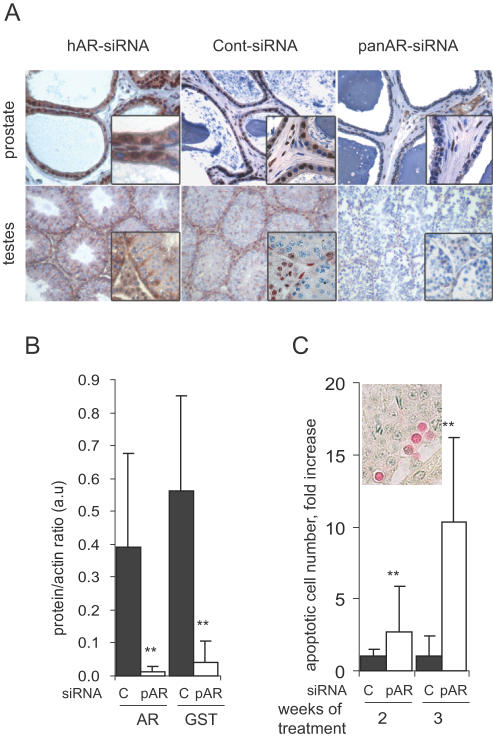
Silencing of AR in prostate and testes. A: Upper panels, immunodetection of AR expression in the ventral prostate of mice treated for 3 weeks with hAR-, cont-, or panAR-siRNA as indicated. Lower panels, AR expression in testes from mice sacrificed at the end of the experiments shown in figure 1C, after 2 weeks of treatment (cont- and panAR-siRNA) or treated for 3 weeks with hAR-siRNA. B: AR and GST expression in testes from mice treated for 3 weeks with cont- (black bars) or panAR-siRNA (pAR, white bars). AR and GST levels were quantified by immunoblot, normalized with actin level, (arbitrary units, mean±SE, *n* = 10). C: Quantification of apoptotic germ cells (insert) in testes collected from mice treated for 2 or 3 weeks (mean number/ 100 seminiferous tubules±SE) with cont- (c) or panAR-siRNA (pAR). **p<0.01 as compared to cont-siRNA treated group.

**Figure 3:**
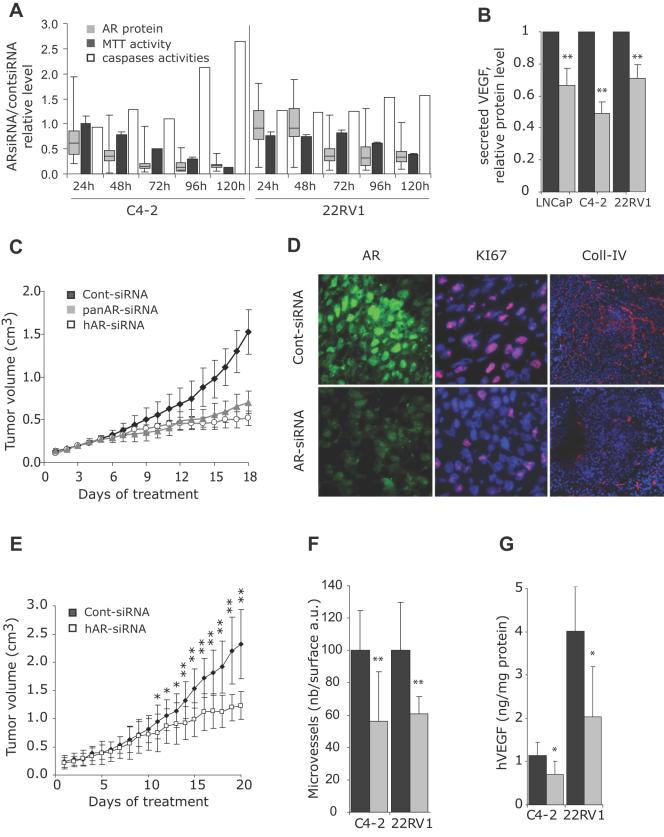
AR-siRNA-induced inhibition of the growth of castration-resistant prostate tumors. A: C4-2 or 22RV1 cells grown in 10% FCS were transfected with 10nM of cont- or panAR-siRNA using HiPerfect reagent. Grey box plots: at the indicated time points, AR was detected by indirect immunofluorescence, and its expression level was quantified in at least 200 nuclei from at least 5 different high power fields using the simple PCI software in cont- or panAR-siRNA transfected cells. Values at each time point were compared to the mean fluorescence value of the cont-siRNA transfected cell population, set to 1. Each box plot is composed of 5 horizontal lines displaying the 10th, 25th, 50th, 75th and 90th percentiles. In sister wells, the MTT activity (black bars) in AR-siRNA treated cells was measured and expressed as a fraction of that of cont-siRNA transfected cells. The measured caspases activities (white bars) were expressed using the following formula: (caspases/MTT in AR-siRNA treated cells)/(caspases/MTT in cont-siRNA treated cells). B: Relative VEGF protein content in the medium of LNCaP, C4-2 or 22RV1 cells, 72 h after transfection of cells with control (dark grey bars) or hAR-siRNA (light grey bars) (mean±SE, *n* = 5 to 7 independent experiments). The medium was not changed after transfection. **p<0.01 as compared to values in cont-siRNA transfected cells set to 1. C: Mice bearing C4-2 vascularized and exponentially growing tumors were randomized and received daily i.p. injections of cont-siRNA (Black symbols), hAR-siRNA (white symbols) or panAR-siRNA (grey symbols); tumor volume (cm^3^, mean±SE *n* = 6-12). *p<0.05 and **p<0.01 comparing panAR-siRNA to cont-siRNA treated tumors (stars above the curves) or comparing hAR-siRNA to cont-siRNA treated tumors (stars below the curves). D: Frozen sections of regions at the periphery of tumors from C were immunostained by indirect immunofluorescence with AR, KI67 or Collagen-IV antibodies and observed by epifluorescence. Photographs were taken with a 63x (AR, KI67) or 10x (Coll-IV) objective, using the same exposure parameters for cont- and AR-siRNA treated tumors. A representative tumor from each group is shown. E: Male mice bearing exponentially growing 22RV1 tumors were randomized and received either cont- (Black symbols) or hAR-siRNA (white symbols); tumor volume (cm^3^, mean±SE, *n* = 5). *p<0.05 and **p<0.01 comparing panAR-siRNA to cont-siRNA treated tumors. F: Mean microvessels density (number of vessels/arbitrary surface unit) in at least 10 non overlapping high-power fields from tumors treated with cont- (black bars) or AR-siRNA (gray bars) in C4-2 or 22RV1 tumors. Necrotic regions were not included in the study. **p<0.01 as compared to cont-siRNA treated group. G: human VEGF quantification in tumor homogenates from C4-2 or 22RV1 tumors in mice treated with cont-siRNA (black bars) or AR-siRNA (gray bars) (ng/mg of protein, mean±SEM). *p<0.05 as compared to cont-siRNA treated group.

In patients, castration or AR antagonists affect not only the tumor cells, but also normal tissues, triggering deleterious side effects. Development of tumor-selective treatments would reduce these unwanted effects but could also be less efficient. To address this question we analyzed the prostate and testes of mice treated for 2 or 3 weeks with daily injections of 3 µg of siRNA. As compared to cont-siRNA, AR expression was strongly reduced by the pan-AR-siRNA treatment in epithelial and stromal cells of the ventral prostate, and in Leydig and Sertoli cells in the testes (Figure 2A). In the testes, the AR-target gene Glutathione S-transferase alpha [26] was also inhibited by AR silencing (Figure 2B). As observed with other AR signaling inhibitors [27], AR silencing in the testes induced the apoptosis of germ cells (Figure 2C), but not that of the AR-expressing Leydig and Sertoli cells, further demonstrating the specificity of the effects triggered by the panAR-siRNA. We recently reported that AR silencing in the testes inhibits the Fibroblast Growth Factor-2 synthesis in the germ cells, a mechanism possibly involved in the induction of their apoptosis [28]. Testosterone is mainly produced by the Leydig cells. We therefore measured whether AR silencing affected the production of this hormone, and found no significant differences between untreated mice (0.88±0.69 ng/ml), and mice treated for 3 weeks with cont-siRNA (0.53±0.31 ng/ml) or panAR-siRNA (0.81±0.52 ng/ml). It is thus unlikely that the antitumoral effects of the panAR-siRNA result from an indirect effect on peripheral tissues. Furthermore, the AR expression in prostate and testes was not affected by treatment of mice with the siRNA selective for the human AR mRNA (Figure 2A) whereas this siRNA efficiently silenced AR in the human tumor cells and inhibited their growth *in vivo* (Figure 1E). In addition, the effects of hAR- and panAR-siRNAs to inhibit the growth of C4-2 tumors were very similar (see below, Figure 3C). Together, these results demonstrate that the main driver of the antitumoral effects of the AR-siRNA is the AR silencing in the tumor cells themselves. Treatment of tumors expressing a mutated AR isoform with siRNAs targeting specifically this mutation would silence AR in the tumor, while preserving its expression is normal tissues, thus reducing the unwanted side effects.

### AR-siRNA directed inhibition of CRCaP growth

The growth of androgen-dependent tumors, such as LNCaP or CWR22, xenografted in mice, is efficiently inhibited by castration. However, tumors eventually adapt to the low androgen environment and recur. The C4-2 [29] and 22RV1 [30] cell lines were respectively isolated from LNCaP or CWR22 recurring tumors and develop in females and in males castrated or treated with bicalutamide [14], [31]. Although C4-2 cells divide in androgen-depleted medium, androgens still efficiently induced the transcription of a 4xARE-luciferase reporter construct (Figure S4A and S4B), confirming that AR is still functional in these cells. However, bicalutamide failed to antagonize the androgen-induced transcription (Figure S4B). In contrast, AR silencing in C4-2 cells, abolished the induction of transcription by androgens (Figure S4B). We then transfected AR-siRNA into C4-2 and 22RV1 cells and compared the kinetics of the AR protein level, the cells' proliferation, quantified by the MTT activity, and apoptosis, measured with a caspases assay were affected. In C4-2 cells, the AR protein level was markedly reduced 2 days after transfection (Figure 3A, grey box plots). Cells' proliferation diminished starting from day 3 (Figure 3A, black bars) and apoptosis increased significantly in C4-2 cells on day 4 and 5 (Figure 3A, white bars). In 22RV1 cells, the AR-siRNA effect appeared less pronounced: the AR protein level was unaffected until day 3 and the proliferation was reduced only after day 4. Moreover, AR silencing did not induce any significant increase in caspases-dependent apoptosis during the 5 first days following transfection (Figure 3A). Therefore, 5 days after transfection, the number of viable cells was higher in 22RV1 than in C4-2 cells.

The Vascular Endothelial Growth Factor (VEGF), which is a key regulator of angiogenesis in the prostate, is upregulated by androgens in the normal prostate and in androgen-dependent cells, through indirect transcriptional [32], [33] or post-transcriptional [34] mechanisms. Although CRCaP tumors are highly angiogenic [35], the role of AR in the control of VEGF production in these tumors was not studied so far. Importantly, we observed that AR silencing reduced the VEGF synthesis in LNCaP as well as in C4-2 and 22RV1 cells (Figure 3B), demonstrating that VEGF is still regulated by AR in advanced prostate cancers.

Our *in vivo* studies using the LNCaP model demonstrated the efficiency and specificity of the antitumoral effects produced by AR silencing. We then studied the effects of the two different AR-siRNAs on the *in vivo* growth of castration-resistant tumors. We first grafted C4-2 cells to nude mice and, after a month, once vascularized tumors were exponentially growing, and reached a mean tumor volume of 129.9±29.1 mm^3^, mice were randomized to receive cont-, or panAR-, or hAR-siRNA. In contrast with castration or bicalutamide, which do not affect the development of C4-2 tumors [36], both the panAR- and the hAR-siRNA efficiently inhibited the C4-2 tumor growth (Figure 3C). In non-necrotic regions, mainly at the periphery of the tumor, a strong reduction in the level of AR expression and in the proportion of KI67-positive proliferating cells was observed (Figure 3D).

Similarly, treatment of mice bearing 22RV1 tumors (mean tumor volume on the day of first siRNA administration: 224.6±104.0 mm^3^) with AR-siRNA markedly repressed the tumor growth (Figure 3E). Despite the presence of large necrotic regions in C4-2 and 22RV1 tumors treated with AR-siRNA, we only seldom observed shrinkage of tumors. This may be due to the strong fibrotic reaction also observed in these tumors. The external volume of the tumors therefore is likely not proportional to the number of viable tumor cells. To further establish that 22RV1 were resistant to castration levels of testosterone and that the AR-siRNA was not dependent on indirect effects on prostate or testes, 22RV1 cells were grafted into female nude mice. Mice bearing exponentially growing tumors with a mean volume of 300 mm^3^ were treated with hAR-siRNA. This treatment inhibited the tumor growth efficiently, although the effect was only visible after a week of treatment (Figure S5).

Importantly, the AR-siRNA treatment of C4-2 and 22RV1 tumors translated into a significant reduction in the number and size of blood vessels (Figure 3D and 3F). This reduction came along with an inhibition of the VEGF production by the human tumor cells (Figure 3G), in agreement with the *in vitro* data (Figure 3B).

### Quantification of siRNA in tumors and mouse tissues

**Figure 4:**
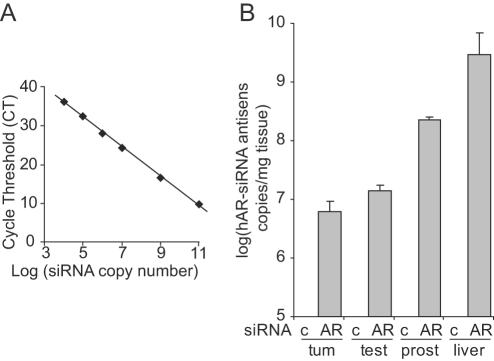
Quantification of siRNA copies in CRCaP tumors and mouse tissues. A: Dynamic range and sensitivity of the hAR-siRNA antisense strand assay. 10^4^ to 10^11^ copies of the antisense strand of hAR-siRNA were reverse-transcribed using a stem-loop primer. The product was amplified in the presence of SyberGreen and the cycle threshold (CT) plotted against the log of the number of copies of the input target. B: Detection of the antisense strand of hAR-siRNA in tumors (tum), testis (test), prostate (prost) and liver of mice treated with 3 µg of cont- (c) or hAR-siRNA (AR) injected i.p. in 50 µl of saline. The number of siRNA copies is normalized to the tissue weight (mean±SE *n* = 3).

To further demonstrate that the antitumoral effects of AR-siRNA in CRCaP resulted from the uptake of the injected naked-siRNA into tumors, we adapted a qRT-PCR method recently set up to quantify miRNAs [37]. A stem-loop primer, with an 8 bases-long overhang complementary to the 3′ end of the hAR-siRNA antisense strand, was annealed at 30°C with known amounts of this antisense strand. Reverse transcription was performed and the product amplified by qPCR. The assay shows an excellent linearity between the log of the number of siRNA copies and the cycle threshold (CT) value, with a dynamic range of at least 7 logs. This method is sensitive enough to detect 10,000 siRNA molecules (Figure 4A). Total RNA extracted from mouse tissues and 22RV1 tumors was then reverse transcribed and amplified, and the number of siRNA molecules was determined by comparison with the standard curve. No signal was amplified in RNA extracted from liver, testes or tumors of mice treated with cont-siRNA (Figure 4B). In contrast, the antisense strand was amplified in tumors, liver, testis and prostate dissected from mice injected with 3 µg of hAR-siRNA (Figure 4B), demonstrating the uptake of naked siRNA injected i.p. This sensitive assay will allow studying further the pharmacokinetics and biodisponibility of siRNA injected in mice (Gilbert et al, in preparation).

## Discussion

Specific gene silencing, and RNA interference in particular, offers new exciting therapeutic perspectives for a number of pathologies, including cancers. In this perspective, RNAi-directed silencing *in vivo* must achieve efficiency, safety and specificity. Infection of cells with lentiviral vectors expressing short hairpin RNA (shRNA), triggers a strong silencing of the target, but therapeutic applications of this approach are limited by risks linked to the stable integration of the viral genome in the host, by the difficulty to infect *in vivo* a large number of cells and by toxic effects resulting from the saturation of the miRNA maturation machinery [38]. We thus chose to use synthetic siRNAs. Mice were treated by daily i.p. injections of small amounts of unmodified siRNAs (125 µg/kg) diluted in saline. We did not observe any change in the weight, behavior and aspect of organs at dissection of mice treated for 3 weeks. As compared to other studies, reviewed in [39], where up to 50 mg/kg of stabilized and/or vectorized siRNAs were used, our settings strongly limit possible toxic or immune effects reported with some chemical modifications of siRNA or vectorization agents [40]. Low siRNA dosing also keeps to a minimum the always possible dose-dependent off-target effects [41].

Several groups injected under a normal pressure small amounts of naked siRNAs into mice and reported the efficient silencing of genes such as VEGF [20], CEACAM6 [42], EphA2 [43], or Bcl2 [44]. Biodistribution studies using radiolabeled naked siRNAs [44], [45] showed that siRNAs rapidly exit the blood compartment and enter into various tissues. However, this technique does not demonstrate the integrity of siRNAs into tissues. We demonstrate, and quantify here for the first time, the uptake of siRNAs into tumors and mouse tissues. The blood vessels density in tumors from AR-siRNA treated mice was low (Figure 3C) and, accordingly, the number of siRNA copies detected in these tumors was of the same order of magnitude than that measured in testes, which are poorly vascularized, whereas the siRNA uptake appeared more efficient in liver and prostate. However the number of siRNA molecules within tumor cells is likely underestimated, due to the heterogeneity of necrotic and fibrotic tumors, the difficulty to quantify the number of viable cells within a tumor, and the presence of nucleases released by dying cells during the RNA purification process. It is still unclear today which transporter, among the several membrane proteins identified [46], is really involved in the uptake of naked oligodeoxynucleotides (ODNs) demonstrated in some clinical trials [47]. Similarly, an homologue of the dsRNA transporter of C. elegans, Sid-1, was recently shown to transport siRNAs into mammalian cells [48]. However, we could not correlate the sid-1 mRNA level with the uptake of siRNA into mouse tissues (data not shown), and it is thus likely that other transporters are also involved. Despite the fact that the molecular mechanism of siRNA uptake into mammalian cells *in vivo* is still under investigation, it is of note that ongoing clinical trials of siRNAs are performed with naked molecules [49].

The role of AR in androgen-dependent prostate carcinomas has been well established over years, and recently confirmed using an inducible AR-shRNA lentiviral construct in LNCaP tumors [50]. We therefore used this cellular model to set up the technical conditions to silence AR in exponentially growing tumors using synthetic AR-siRNA. The uptake of panAR-siRNA triggered a strong silencing of AR in tumor cells, prostate and testes which did not modify the blood level of testosterone. Interestingly, the sequence specificity of siRNA allowed here discriminating *in vivo* the closely related mouse and human AR mRNAs: treatment with hAR-siRNA silenced AR in tumor cells and inhibited the tumor growth while preserving AR expression in mouse prostate and testes. Treating tumors with hAR-siRNA was as efficient as treating with the antagonist bicalutamide, which affects AR signaling in prostate and testes. The inhibition of the growth of C4-2 tumors by panAR- or hAR-siRNAs in intact males was comparable. All together, these data demonstrate that the inhibition of AR signaling outside the tumor does not participate in the antitumoral effect, opening promising perspectives for developing tumor-specific treatments with reduced side effects for patients.

Prostate tumor cells can escape androgen-ablation therapies by multiple mechanisms involving the androgen receptor. The functionality of AR in these resistant cells is well established, but this does not foretell if AR silencing is sufficient to inhibit proliferation and trigger apoptosis, as other molecular events accumulating along tumor progression could help cells bypassing AR signaling. Of note, DU145 and PC3 cell lines, which no longer express AR, are representative of a proportion of advanced prostate tumors where AR is no longer required for tumor growth [11]. Several studies evaluated the role of AR in CRCaP cells *in vitro*, leading to contradictory conclusions: Gosh et al., showed that the main driver of CRCaP cells' division was p70S6 kinase, and not AR [14] and two studies showed that RNAi-induced AR silencing in these cells produced only a limited [15] or a non statistically significant effect [16]. In contrast, Zegarra-Moro et al. [13] showed that the microinjection of AR antibodies almost completely arrested C4-2 cells' proliferation, whereas Liao et al. [17] reported that AR silencing induced their massive apoptosis. In our hands, AR silencing produced a time-dependent inhibition of cells' proliferation in C4-2 as in 22RV1 cells, but triggered apoptosis in C4-2 cells only, at least during the time-course of this experiment. Using a lentiviral AR-shRNA expressing vector, Chen et al [6] stably silenced AR in LAPC4 and in castration-resistant LNCaP cells, which then failed to develop tumors once grafted into SCID mice. However, as the authors did not report if the cells' proliferation was affected or not before the grafting, the *in vivo* data are difficult to interpret. From all the above studies, it was therefore not possible to foretell whether AR was a key target in established resistant tumors.

We used to address this question one ADCaP and two unrelated CRCaP models and checked during at least 5 days that each tumor was exponentially growing before randomization for siRNA treatment. AR-siRNA induced in all three tumor models a strong inhibitory effect. The tumors that were smaller at the start of the treatment (for example 50 mm^3^ in a 24 g-weighing mouse in Figure 1C, which would correspond to a tumor of 146 g in a 70 kg-weighing man) responded better than the larger ones.

It is well established that AR regulates the VEGF production in the normal prostate as in androgen-dependent tumors. The VEGF level is very tightly controlled, at the transcriptional and post transcriptional levels, and AR is likely involved in several of these mechanisms [32], [33], [34]. Tumors that became resistant to hormonal manipulations are very angiogenic [35] and express high VEGF levels. Importantly, we demonstrate here for the first time that, *in vitro* as *in vivo*, AR still controls the VEGF production in CRCaP. AR silencing *in vivo* reduces VEGF synthesis, triggering a reduction in the blood vessels density, although other modulators of angiogenesis regulated by androgens [35], [51], [52] could also participate in this process. Therefore, part of the antitumoral effects produced by AR-siRNA likely result from the inhibition of angiogenesis in the treated tumors. Our results demonstrate that androgen dependent, but also and more importantly, exponentially growing and vascularized castration-resistant prostate tumors, are still dependent on the expression of a functional AR for their growth and angiogenesis.

Importantly, siRNA-directed silencing of AR allows inhibiting the synthesis of overexpressed wild type receptors, as well as mutated or stabilized isoforms that can be encountered in advanced prostate carcinomas. The strong antitumoral effects triggered by siRNA-directed silencing of AR, which results from a combination of antiproliferative and antiangiogenic effects, opens exciting therapeutic perspectives for treating advanced prostate tumors that became resistant to hormonal manipulations, for which treatments available today are only palliative.

## Materials and Methods

### Cell culture and in vitro assays

LNCaP, 22RV1 and C4-2 cells were grown in RMPI containing 10% of fetal calf serum. *In vitro* transfections of plasmids and siRNA (10 nM) were performed by a standard calcium phosphate precipitation method or, in Figure 3A, using the HiPerfect reagent (Qiagen, Courtaboeuf, France). Androgen reporter assays were performed as previously described [35]. When stated, cells were incubated in medium containing 10% charcoal-stripped fetal calf serum and then treated with R1881, a synthetic AR-specific agonist (0.1–0.5 nM, NEN, Zaventem, Belgium), or with the AR-antagonist bicalutamide (100 nM, generous gift of AstraZeneca) or with vehicle. Cell proliferation and viability were quantified either by counting cells excluding the trypan blue or using the MTT assay (WST-1, Roche). Caspases activities were measured using a fluorimetric quantification assay (Apo-One caspase 3/7 assay, Promega, Lyon France).

### siRNA

Synthetic siRNA were purchased from Dharmacon (option A4, Boulder, Co). Sequences are indicated in table S1.

### Animals, siRNA injection and tumorigenicity assays

Studies involving animals, including housing and care, method of euthanasia and experimental protocols were conducted in accordance with the local animal ethical committee in the Institut André Lwoff in Villejuif, France. Tumor cells (2×10^6^ cells/mouse) were injected subcutaneously in 50% (v:v) matrigel (BD biosciences) to 6–8 weeks old male nude mice and measured as previously described [35]. The tumor take was about 65% for LNCaP cells, and 80% for C4-2 and >90% , in males as in females, for 22RV1 cells. When indicated, 50 µl of a 4.2 µM solution of siRNA diluted in PBS were injected i.p. on a daily basis. Bicalutamide (50 mg/kg/day, Casodex, AstraZeneca) was administered orally.

### Real-time RT-PCR siRNA and mRNA analysis

Total RNA was isolated using TRIzol reagent (Invitrogen, Cergy Pontoise, France). Relative gene expression was analyzed using QuantiTect SYBR Green RT-PCR kit (QIAGEN, Courtaboeuf, France). Human Cyclophilin A was used as an internal control. For siRNA detection, 500 ng of total RNA was annealed at 30°C for 30 min with a stem-loop primer and then reverse transcribed (Taqman microRNA reverse transcriptase kit, Applied). The RT product was then amplified, using the same kit as above. Sequences of primers are indicated in table S2. Threshold cycle (CT) values, defined as the fractional cycle number at which the fluorescence passes the fixed threshold, were converted into absolute copy numbers using a standard curve from synthetic hAR-siRNA antisense strand.

### Immunodetection and apoptosis detection

Protein detection by immunoblotting was performed using antibodies raised against AR (rabbit polyclonal antibody sc-816 Santa Cruz, CA), Gluthation-S Transferase alpha 4 (NCL-GSTalpha, Novocastra-Tebu, Le Perray en Yvelines, France), actin (clone AA 20–33, Sigma, Meylan, France) or tubulin (mouse monoclonal DM1A, Sigma). Vascular Endothelial Growth Factor (VEGF) was quantified using an ELISA kit specific for human VEGF (R&D Systems, Minneapolis, MN, USA). Testosterone in blood samples was quantified by RIA (Beckman-Coulter, Villepinte, France). Immunodetection on paraffin embedded tissues or cryostat sections was performed using antibodies raised against AR (rabbit polyclonal: Santacruz sc-816 or mouse monoclonal: Neomarkers Ab1), KI67 (Neomarkers, rabbit monoclonal) to label proliferative cells or Collagen IV (Novotec, Lyon, France) to detect microvessels. TUNEL labeling was performed as previously described [53].

### Statistical analysis

Using a t-test, results were considered statistically different when calculated p values were <0.05 (*) or <0.01 (**).

## Supporting Information

Figure S1 Control of the exponential growth of tumors before first treatment. Before randomization of mice for siRNA treatment, the exponential growth of tumors was verified by plotting the neperian logarithm of the tumor volume as a function of time. This analysis was performed for each individual tumor studied. The figure represents the ln(tumor volume), mean±SEM, of tumors studied in figure 1C, before the first siRNA treatment.
(0.53 MB TIF)

Figure S2 Effect of panARsiRNA treatment on the growth of an AR-negative tumor . JT8 fibrosarcoma cells, which do not express AR, were grafted to male nude mice. Starting from day 8, mice received daily i.p. injections of 3 µg of cont-siRNA (black symbols) or panAR-siRNA (white symbols). The tumor volume (mean±SE, n = 6) was not different between the two groups. VEGF-siRNA injected with the same protocol were shown previously to induce 70% reduction in VEGF production and a marked growth inhibition of these tumors (Filleur et al., cancer res. 2003).
(0.61 MB EPS)

Figure S3 Naked siRNAs do not induce in mice a typeI-Interferon response. Interferon-a was quantified by ELISA (R&D) in serum collected from mice bearing LNCaP tumors and treated daily for 15–21 days with 3 µg of either cont-siRNA, hAR-siRNA or panAR-siRNA (experiments shown in Fig 2a and 3a). Similar results were obtained when mice were treated with siRNA for only 3 days (not shown). As controls, naive mice were injected i.p. for 2 consecutive days with 80 µg or 3 µg of poly[I ]-poly[C], or with 80 µg of poly[I ]-poly[C] pretreated with RNAse A. Results are expressed in pg/ml of serum on a logaritmic scale.
(0.61 MB EPS)

Figure S4 Effects of bicalutamide and siRNA on LNCaP and C4-2 cells in vitro. A: LNcaP (grey bars) or C4-2 (black bars) cells were incubated for 3 days in medium containing 10% of charcoal-filtered serum supplemented with R1881 (0.5 nM) or bicalutamide (10-5M) as indicated. The cell number was compared to that of untreated cells. B: A 4xARE-luciferase reporter gene was co-transfected into LNCaP (grey bars) or C4-2 cells (black bars) along with cont- or hAR-siRNA. Cells were then incubated in medium containing 10% of charcoal-filtered serum supplemented with R1881 (0.5 nM) or bicalutamide (10-5M) as indicated. The luciferase activity was quantified 48 h after transfection.
(0.72 MB EPS)

Figure S5 Inhibition of the 22RV1 tumor growth in female mice by hAR-siRNA treatment. 6 weeks-old female swiss nu/nu mice were grafted with one million 22RV1 cells. Once tumors grew exponentially, mice were randomized (6 mice per group) and received daily i.p. injections of 3 µg of cont-siRNA or hAR-siRNA diluted in PBS.
(0.50 MB EPS)

Table S1 (0.03 MB DOC)

Table S2 (0.03 MB DOC)
