# Supplementary material for: SIRNA-Directed In Vivo Silencing of Androgen Receptor Inhibits the Growth of Castration-Resistant Prostate Carcinomas
Source: PLoS One. 2007 Oct 10;2(10):e1006. doi: 10.1371/journal.pone.0001006 (PMC1994591; doi:10.1371/journal.pone.0001006)
Supplement: Table S1 — (0.03 MB DOC) [file pone.0001006.s006.doc]

**Table S1: Sequences of siRNA used**

| siRNA | Sequence of the sense strand (5'-3') |
| --- | --- |
| panAR-siRNA | GACUCAGCUGCCCCAUCCA-dTdT |
| hAR-siRNA | UCCcCAAGCCCAucguAGA-dTdT  (bases in lower case differ from the mouse AR sequence) |
| Cont-siRNA | GAUAGCAAUGACGAAUGCGUA-dTdT |
