# Supplementary material for: SIRNA-Directed In Vivo Silencing of Androgen Receptor Inhibits the Growth of Castration-Resistant Prostate Carcinomas
Source: PLoS One. 2007 Oct 10;2(10):e1006. doi: 10.1371/journal.pone.0001006 (PMC1994591; doi:10.1371/journal.pone.0001006)
Supplement: Table S2 — (0.03 MB DOC) [file pone.0001006.s007.doc]

**Table S2: Sequences of primers used**

| mRNA | Primer sequence (5'-3') |
| --- | --- |
| Human Androgen Receptor (AR) | Forward: GGAACAGCAACCTTCACAGC  Reverse: CTTTAAGGTCAGCGGAGCAG |
| Human Prostate Specific Antigen (PSA) | Forward: ACCAGAGGAGTTCTTGACCCCAAA  Reverse: CCCCAGAATCACCCGAGCAG |
| Human Vascular Endothelial Growth Factor (VEGF) | Forward: GGACATCTTCCAGGAGTACC  Reverse: TCCGCATAATCTGCATGGTG |
| Human cyclophilinA | Forward: CCCATTTGCTCGCAGTATCCTAGA  Reverse: GGCATGGGAGGGAACAAGGAAAAC |
| hAR-siRNA amplification | Stem-loop RT primer:  GTCGTATCCAGTGCAGGGTCCGAGGTATTCGCACTGGATACGACAATCCCCA  Forward: CGCGCTCTACGATGGGC  Reverse: GTGCAGGGTCCGAGGT |
